# Chemo-enzymatic synthesis of tetrasaccharide linker peptides to study the divergent step in glycosaminoglycan biosynthesis

**DOI:** 10.1093/glycob/cwae016

**Published:** 2024-02-24

**Authors:** Marie Bourgeais, Farah Fouladkar, Margot Weber, Elisabetta Boeri-Erba, Rebekka Wild

**Affiliations:** Institut de Biologie Structurale, UMR 5075, University Grenoble Alpes, CNRS, CEA, 71, Avenue des Martyrs, 38044 Grenoble, France; Institut de Biologie Structurale, UMR 5075, University Grenoble Alpes, CNRS, CEA, 71, Avenue des Martyrs, 38044 Grenoble, France; Institut de Biologie Structurale, UMR 5075, University Grenoble Alpes, CNRS, CEA, 71, Avenue des Martyrs, 38044 Grenoble, France; Institut de Biologie Structurale, UMR 5075, University Grenoble Alpes, CNRS, CEA, 71, Avenue des Martyrs, 38044 Grenoble, France

**Keywords:** chemo-enzymatic synthesis, glycosaminoglycans, glycosyltransferase enzymes

## Abstract

Glycosaminoglycans are extended linear polysaccharides present on cell surfaces and within the extracellular matrix that play crucial roles in various biological processes. Two prominent glycosaminoglycans, heparan sulfate and chondroitin sulfate, are covalently linked to proteoglycan core proteins through a common tetrasaccharide linker comprising glucuronic acid, galactose, galactose, and xylose moities. This tetrasaccharide linker is meticulously assembled step by step by four Golgi-localized glycosyltransferases. The addition of the fifth sugar moiety, either N-acetylglucosamine or N-acetylgalactosamine, initiates further chain elongation, resulting in the formation of heparan sulfate or chondroitin sulfate, respectively. Despite the fundamental significance of this step in glycosaminoglycan biosynthesis, its regulatory mechanisms have remained elusive. In this study, we detail the expression and purification of the four linker-synthesizing glycosyltransferases and their utilization in the production of fluorescent peptides carrying the native tetrasaccharide linker. We generated five tetrasaccharide peptides, mimicking the core proteins of either heparan sulfate or chondroitin sulfate proteoglycans. These peptides were readily accepted as substrates by the EXTL3 enzyme, which adds an N-acetylglucosamine moiety, thereby initiating heparan sulfate biosynthesis. Importantly, EXTL3 showed a preference towards peptides mimicking the core proteins of heparan sulfate proteoglycans over the ones from chondroitin sulfate proteoglycans. This suggests that EXTL3 could play a role in the decision-making step during glycosaminoglycan biosynthesis. The innovative strategy for chemo-enzymatic synthesis of fluorescent-labeled linker-peptides promises to be instrumental in advancing future investigations into the initial steps and the divergent step of glycosaminoglycan biosynthesis.

## Introduction

Glycosaminoglycans (GAGs) are intricate and long polysaccharides covalently bound to diverse core proteins, collectively forming proteoglycans (PGs). These molecular entities are ubiquitous in nearly all metazoan organisms (Yamada et al. 2011). Depending on their disaccharide unit and modifications, the attached polysaccharide chains categorize into distinct types, including heparan sulfate (HS), heparin (Hep), chondroitin sulfate (CS), dermatan sulfate (DS), and keratan sulfate (KS) (Merry et al. 2022). Despite the seemingly limited varieties of Heparan Sulfate Proteoglycans (HSPGs) (20) and Chondroitin Sulfate Proteoglycans (CSPGs) (80), they have been found to interact with an astonishingly extensive array of protein partners, numbering over 3,000 to date (Vallet et al. 2022). These interaction partners include growth factors, cytokines, morphogens, signaling receptors, chemokines, and pathogens, implicating them in a myriad of biological processes (Bishop et al. 2007; Connell et al. 2016). This expansive interactome is mirrored by the intricate structural complexity of GAG chains, which poses a considerable challenge in identifying specific GAG-protein interactions (Annaval et al. 2020). The imperative for a more profound understanding is underscored by the involvement of proteoglycans in diverse pathological processes, spanning from cancer and inflammation to Alzheimer’s disease and pathogen infections (Iozzo and Sanderson 2011; Connell and Lortat-Jacob 2013; Gschwandtner et al. 2017; Gopal 2020; Ozsan McMillan et al. 2023). Yet, despite these critical roles, the availability of drugs targeting proteoglycans remains limited, likely due to the lack of a detailed understanding of the structure-function relationship (Morla 2019; Theocharis et al. 2019). Thus, there is a strong need for innovative methodologies to probe GAG biosynthesis, comprehend its regulation, and facilitate the development of GAG mimetics with precisely defined sequences.

The biosynthesis of Hep, HS, CS and DS commences with the step-wise addition of a common [GlcAβ1–3Galβ1–3Galβ1–4Xyl] tetrasaccharide linker onto the serine residue of the core protein. This process takes place in the Golgi lumen, facilitated by the peptide-O-xylosyltransferases, XylT1/XylT2, which transfer a xylose (Xyl) from UDP-Xyl onto the side chain of the serine residue (Götting et al. 2000; Pönighaus et al. 2007). The crystal structure of XylT1, in complex with acceptor peptide substrates, has revealed the specific recognition mechanism of the Ser-Gly motif (Briggs and Hohenester 2018). Subsequently, a galactose (Gal) molecule is transferred from a UDP-Gal substrate by β4GalT7 (Almeida et al. 1999; Tsutsui et al. 2013), followed by the addition of a second Gal molecule through the action of β3GalT6 (Bai et al. 2001; van Damme et al. 2018). Linker synthesis is completed by the glucuronyltransferase GlcAT-1 catalyzing the transfer of glucuronic acid (GlcA) from UDP-GlcA (Pedersen et al. 2000, 2002; Kitagawa et al. 2001).

The addition of the fifth sugar molecule dictates the fate of the resulting glycosaminoglycan chain. Transfer of a N-acetylglucosamine (GlcNAc) molecule by EXTL3 or EXTL2 leads to the formation of Hep/HS chains (Kitagawa et al. 1999; Kim et al. 2001; Nadanaka et al. 2013; Wilson et al. 2022). In contrast, N-acetylgalactosamine (GalNAc) transfer by CSGALNACT-1 or CSGALNACT-2 directs subsequent biosynthesis towards the generation of CS/DS chains (Sato et al. 2003; Gulberti et al. 2012). The regulatory mechanism governing the addition of either GlcNAc or GalNAc, thus determining the formation of HSPG or CSPG respectively, remains enigmatic.

One conceivable mechanism posits that HSPGs and CSPGs exhibit distinct, yet conserved features in their amino acid sequence and/or three-dimensional structure. These features might be specifically recognized by the EXTL3 or CSGALNACT-1/CSGALNACT-2 enzymes. Previous bioinformatic studies have indeed revealed conserved attributes in the amino acid sequence surrounding the glycosaminoglycan attachment site: (i) an enrichment of serine and glycine residues, alongside large, hydrophobic residues in close proximity to the attachment site in HSPGs, and (ii) an accumulation of negatively charged residues in CSPGs (Zhang et al. 1995; Briggs and Hohenester 2018; Toledo et al. 2020). Despite these characteristics, accurately predicting whether a given protein contains a GAG chain remains challenging. Additionally, predicting the specific type of GAG chain that will be added remains impossible.

Thus, there is a strong need for innovative tools that facilitate a more detailed investigation of the divergent step in GAG biosynthesis. In this study, we present the chemo-enzymatic synthesis of fluorescent-labeled peptides bearing the native tetrasaccharide linker, achieved through the use of recombinantly expressed and purified linker-synthesizing glycosyltransferases (GTs). A similar approach was recently described by Sammon et al. (Sammon et al. 2023). This methodology will significantly advance future studies aimed at characterizing both the initial and the divergent steps of GAG biosynthesis.

## Materials and methods

### Construction of expression vectors

#### XylT1

The coding sequence for human XylT-I (Uniprot #Q86Y38), encompassing residues 232 to 959, was synthesized with an N-terminal BM40 signal sequence, an 8xHistidine tag, and a TEV cleavage site. This construct was then inserted into the pTWIST-CMV-WPRE-Neo plasmid (Twist Bioscience).

#### β4GalT7

The coding sequence for human β4GalT7 (Uniprot #Q9UBV7), spanning residues 81–327, was cloned utilizing *NcoI* and *NotI* restriction sites into the pETM30 vector backbone. This vector includes an N-terminal 6xHistidine tag, a GST, and a TEV site cleavage (Chatron-Colliet et al. 2017).

#### β3GalT6

The coding sequence for human β3GalT6 (Uniprot #Q96L58), ranging from residues 53–329, was synthesized (Twist Bioscience) and integrated into the pHR-CMV-TetO2 vector using *BshTI* and *KpnI* restriction sites. This resulted in an N-terminal chicken RPTPσ signal sequence, followed by a SUMO fusion protein, a 6xHistidine tag, and a 3C cleavage site (Elegheert et al. 2018; Behiels and Elegheert 2021).

#### GlcAT-1

The coding sequence for human GlcAT-1 (Uniprot #O94766), spanning residues 76 to 335, was custom-synthesized for bacterial expression (Twist Bioscience). It was then cloned into the pETM30 vector using *NcoI* and *NotI* restriction sites. This construct featured an N-terminal 6xHistidine tag, a GST tag, and a TEV cleavage site.

#### EXTL3

The coding sequence for human EXTL3 (Uniprot #O43909), ranging from residues 53 to 919, was custom-synthesized (Twist Bioscience) and integrated into the pHR-CMV-TetO2 vector using *BshTI* and *KpnI* restriction sites. The resultant vectors encoded an N-terminal chicken RPTPσ signal sequence, followed by a SUMO fusion protein, a 6xHistidine tag, and a 3C cleavage site.

### Protein expression and purification

#### XylT1

Two batches of 225 mL of 293-F cells at a concentration of 1.10^6^ cells/mL were prepared in FreeStyle™ 293 medium (Thermo Fisher). Cultures were incubated with agitation at 37 °C in a 5% CO_2_ environment for all described steps. Transfections with the pTWIST-Xylt1_232-959 were performed 24 h later, once cultures reached approximately 2.10^6^ cells/mL, using a mass ratio of polyethylenimine (PEI) to DNA in a 2:1 ratio (500 μg PEI: 250 μg DNA) and resulting in a total culture volume of 250 mL per Erlenmeyer flask. After 96 h of incubation, cell cultures were centrifuged for 5 min at 500 × *g*. The resulting supernatants were supplemented with protease inhibitor cocktail tablets (Roche) and further centrifuged for 15 min at 6,000 × *g* at 4 °C. The supernatants were then filtered through a Durapore 0.45 μm PVDF membrane (Merck Millipore) and 50 mM Tris (pH 7.4), 250 mM NaCl, 10 mM MgCl_2_, and 10 mM imidazole were added. The protein suspension was loaded onto a HisTrap HP™ 5 mL column (Cytiva) that had been pre-equilibrated with Tris buffer A (50 mM Tris, pH 7.4, 250 mM NaCl). Elution was carried out using a gradient of imidazole concentrations (20 mM, 50 mM, 100 mM, 200 mM, 500 mM, and 750 mM) achieved by mixing Tris buffer A and Tris buffer B (50 mM Tris, pH 7.4, 250 mM NaCl, 1 M imidazole, and 5% glycerol (v/v)). All elution fractions were collected and subsequently analyzed by SDS-PAGE. Fractions containing the protein of interest were pooled and transferred into a dialysis membrane with a molecular weight cut-off of 3.5 kDa (SpectraPor ®3 Dialysis Membrane Standard Rc Tubing, Spectrum Labs™). The protein was dialyzed against 2 L of Tris buffer A containing 5% glycerol for 16 h at 4 °C. The resulting supernatant was concentrated to a final concentration of 0.5 mg/mL (total yield of 1.7 mg) using a 30 kDa molecular weight cut-off centrifugal filter (Amicon Ultra, Millipore, Sigma-Aldrich). Aliquots were prepared, snap-frozen in liquid nitrogen, and stored at −80 °C.

#### β4GalT7

The pETM30-β4GalT7_81-327 plasmid was transformed into *E. coli* Rosetta(DE3)pLysS bacterial cells. Four liters of Luria-Bertani broth medium supplemented with kanamycin (15 μg/mL) and chloramphenicol (34 μg/mL) were inoculated with a preculture and cultivated at 37 °C with shaking. Cultures were shifted to 16 °C upon reaching an OD value of 0.2 at 600 nm and induced with 0.25 mM IPTG when the OD value reached 0.6 at 600 nm. Incubation was extended overnight at 16 °C. Bacteria were pelleted at 6,000 × *g* for 15 min and washed with 100 mL of PBS. Following an identical centrifugation step, pellets were resuspended in 120 mL of lysis buffer, composed of 25 mM HEPES (pH 7.4), 500 mM NaCl, 1 M urea, 5% glycerol (v/v), 10 mM imidazole, 200 U of DNAse I (Roche), and 2 protease inhibitor tablets (EDTA-free, Roche cOmplete). Cells were lysed by sonication on ice (using a 4 min program of 2 s on, 8 s off). The resulting lysate was diluted two times in cold lysis buffer, spun down at 35,000 × *g* for 30 min at 4 °C, and the supernatant was sterile-filtered. All subsequent purification steps were conducted at 4 °C and analyzed by SDS-PAGE. The supernatant was applied to a HisTrap HP™ 5 mL column (Cytiva) pre-equilibrated with HEPES buffer (25 mM HEPES, pH 7.4, containing 500 mM NaCl and 5% glycerol (v/v)). After washing with HEPES buffer containing 200 mM imidazole, β4GalT7 was eluted with 500 mM imidazole in HEPES buffer. Two milligrams of His-tagged Tobacco Etch Virus (TEV) protease were added, and reactions were dialyzed overnight against 4 L of HEPES buffer. The supernatant was reloaded onto the HisTrap HP™ column and cleaved β4GalT7 eluted at 50 mM imidazole. Pooled fractions were concentrated using a 30 kDa molecular weight cut-off centrifugal filter (Amicon Ultra, Millipore, Sigma-Aldrich) and injected onto a Superdex 200 Increase 10/300 GL (Cytiva) pre-equilibrated in S200 Buffer (25 mM MES, pH 6.5, 200 mM NaCl, 5% glycerol (v/v)). Elution fractions containing pure β4GalT7 were pooled, concentrated to 4.9 mg/mL (total yield of 3.6 mg), and snap-frozen for storage at −80 °C.

#### SUMO-β3GalT6

Transfection of 600 mL of 293-F cells with the pHR-β3GalT6_53-329 plasmid was carried out, followed by a one-step IMAC purification procedure, similar to the method employed for XylT1 as described above. The purified protein was dialyzed against Tris buffer A supplemented with 5% glycerol (v/v). Subsequently, the protein was concentrated to a final concentration of 2.8 mg/mL (total yield of 2.1 mg). The concentrated protein was then snap-frozen in liquid nitrogen and stored at −80 °C for future use.

#### GlcAT-1

The pETM30-GlcAT-1_76-335 plasmid was transformed into competent *E. coli* BL21(DE3)pLysS bacterial cells. Protein purification followed a protocol similar to that employed for β4GalT7, as described above, with a minor deviation. After removing the 6xHis-GST tag, the protein was retrieved in the flow-through of the second IMAC step. Subsequently, the protein was concentrated to 1.8 mg/mL and snap-frozen for storage (total yield of 1.4 mg).

#### EXTL3

Transfection of 600 mL of 293-F cells with the pHR-EXTL3_53-919 plasmid, followed by the initial IMAC purification step, was conducted in a manner analogous to the procedure employed for XylT1 as described above. Subsequently, 2 mg of His-tagged 3C protease were introduced, and the reactions were dialyzed overnight against 2 L of Tris buffer A supplemented with 5% glycerol (v/v). The resulting supernatant was reloaded onto the HisTrap HP™ column, and cleaved EXTL3 was eluted with Tris buffer A containing 25 mM imidazole. Pooled fractions were concentrated and simultaneously buffer exchanged into Tris buffer A with 5% glycerol (v/v) using a 30 kDa molecular weight cut-off centrifugal filter (Amicon Ultra, Millipore, Sigma-Aldrich). This yielded a final concentration of 5.4 mg/mL, totaling 2.5 mg. The concentrated protein was promptly snap-frozen in liquid nitrogen and stored at −80 °C for future use.

### Nano differential scanning fluorimetry measurements

Thermal stability of purified XylT1, β4GalT7, SUMO-β3GalT6, and GlcAT-1 were assessed using nano differential scanning fluorimetry technology (nanoDSF). Protein samples were diluted to a concentration of 0.3 mg/mL in their corresponding storage buffers: XylT1 and SUMO-β3GalT6 in Tris A buffer with 5% glycerol (v/v), β4GalT7 in S200 buffer, and GlcAT-1 in HEPES A buffer. Standard-grade glass capillaries (Nanotemper) were filled with 15 μL of protein suspension and loaded onto the tray of Prometheus NT.48 instrument (NanoTemper Technologies). The experiment was run using a 1.5 °C per minute thermal ramp over a temperature range from 25 °C to 95 °C at 80%–100% excitation power. Acquisition was followed by data analysis using the PR.ThermControl v.2.3.1 software in order to establish melting temperatures for each sample.

### Mass photometry analysis

Mass photometry analyses were conducted using the Refeyn OneMP device (Oxford, UK). Purified proteins were diluted in the following buffers: XylT1 and SUMO-β3GalT6 in Tris buffer A with 5% glycerol (v/v), β4GalT7 in S200 buffer, and GlcAT-1 in HEPES buffer. Cover slides (24×50 mm, 170 μm) were thoroughly rinsed with demineralized water and isopropanol, and subsequently dried under a clean stream of compressed air. A reusable culture well gasket (Grace Bio-Labs) was placed onto a cover slide. To establish focus, 19 μL of buffer was added to the well and autofocus mode was engaged using the Refeyn AcquireMP v.2.4.0 software. Movies, consisting of 60 s (6,000 frames), were recorded with a standard field-of-view, and the data was processed using DiscoverMP v2.5.0 software. Calibration was performed by adding 1 μL of 1:20 diluted NativeMark Protein Standard (Thermo Fisher Scientific) in buffer. For each acquisition, either 18 μL or 19 μL of buffer were employed to set the focus, followed by the addition of 1 μL to 2 μL of the protein sample to achieve a final concentration between 50 nM and 125 nM. Duplicates were carried out for each sample.

### Tetrasaccharide linker synthesis

Peptide sequences GGPSGDFE (SDC3_short), VSGGPSGDFELPEEET (SDC3_long), GDSSGWPD (TGFBR3), EEASGEAS (CSF1_short) and VPEEASGEASEIPVPQ (CSF1_long) were synthesized with an additional N-terminal TAMRA fluorophore (SB-PEPTIDE, SmartBioscience). Peptides SDC3_short, SDC3_long and TGFBR3 peptides were dissolved in 30% acetonitrile (v/v), while peptides CSF1_short and CSF1_long were suspended in demineralized water, resulting in a final concentration of 1 mM for each. All enzymatic assays were conducted at room temperature in the dark, using a reaction buffer consisting of 25 mM MES pH 6.5, 125 mM NaCl, 2.5 mM MgCl_2_, and 2.5 mM MnCl_2_. For the first condition, a mixture of 100 μM of peptide, 1 mM UDP-Xyl, and 1 μM XylT1 was prepared. A second reaction was set up similarly, but with the addition of 1 mM UDP-Gal and 8.4 μM β4GalT7. Both mixtures were incubated for 16 h and then stored at −20 °C to halt the reactions. Reaction three was prepared as the second reaction, but 1 mM UDP-Gal and 3.15 μM SUMO-β3GalT6 were added after the initial 16 h incubation. Reaction four was set up as reaction 3, and 1 mM UDP-GlcA and 3.15 μM GlcAT-1 were simultaneously added with SUMO-β3GalT6. Reactions three and four were incubated for an additional 24 h before being stored at −20 °C. To analyze the glycan transfer on the peptides, gel electrophoresis was conducted. One microliter of each reaction was mixed with 39 μL of 20% glycerol (v/v) containing 1% phenol red (v/v), and 5 μL of the mixture were loaded onto a 25% polyacrylamide gel (not containing any sodium dodecyl sulfate (SDS)). Migration was carried out in a Tris-glycine running buffer, also lacking SDS. Visualization was achieved using a ChemiDoc MP instrument with Alexa 546 detection settings. The experiment was repeated three times for each peptide. Two identical replicate mixtures were pooled, adjusted to 200 μL with Tris A buffer, and injected onto two Superdex Peptide 10/300 GL (Cytiva) columns connected in line, pre-equilibrated with Tris buffer A.

### Purification of tetrasaccharide linker peptides

For the larger-scale synthesis of the tetrasaccharide linker on peptides, a 300 μL reaction mixture was prepared, consisting of 100 μM of a specific peptide, 1 mM UDP-Xyl, 1 mM UDP-Gal, 1 μM XylT1, and 8.4 μM β4GalT7 in reaction buffer (25 mM MES pH 6.5, 125 mM NaCl, 2.5 mM MgCl_2_, and 2.5 mM MnCl_2_). Following a 16 h incubation at room temperature in the dark, an additional 1 mM UDP-Gal, 1 mM UDP-GlcA, 3.15 μM SUMO-β3GalT6, and 3.15 μM GlcAT-1 were introduced, bringing the final volume to 366 μL. Incubation was extended for an additional 24 h, and the reactions were halted by transferring the mixture to −20 °C. The entire sample was then injected onto two Superdex Peptide 10/300 GL columns (Cytiva) connected in line, pre-equilibrated with Tris buffer A. Elution fractions of the SDC3_long peptide were analyzed by polyacrylamide gel electrophoresis. Peak fractions of the different glycosylated peptides were pooled and dialyzed using a dialysis membrane with a molecular weight cut-off of 1 kDa (SpectraPor). This dialysis was carried out against 4 L of water at 4 °C in the dark for 3 h. A second dialysis step was performed overnight. The samples were then lyophilized using an Alpha 1–2 LD plus instrument (Martin Christ). Lyophilisates were dissolved in demineralized water and their concentrations were determined using titration curves prepared with non-glycosylated peptides. Absorbance values were measured at 554 nm using a Nanodrop 2,000 (Thermo Scientific). Peptides carrying the full linker were stored at −20 °C.

### Mass spectrometry analysis

A commercial Matrix Assisted Laser Desorption Ionisation (MALDI)-time-of flight (TOF)/TOF mass spectrometer (Autoflex MAX, Bruker Daltonics, Bremen, Germany) was used for the measurements. Ionization was achieved using a Bruker-proprietary (Nd:YAG) 355 nm smartbeamTM II solid state laser (maximal frequency: 2 kHz). The laser energy was just above the threshold required for the formation of ions. The measurements of peptides were performed in reflector positive ion mode, detecting 700–5,000 m/z range. Each mass spectrum was the average of 10,000 laser shots randomly acquired at different sample area. We used Bruker AnchorChip™ plate as target. We diluted unmodified peptide (15 μM) 1:3 using 5% formic acid (FA) solution to obtain a 5 μM solution. The glycosylated peptide was not diluted (final concentration: 5 μM). We used a procedure to deposit samples on the MALDI plate as described previously (Signor and Boeri Erba 2013). Briefly, we utilized a thin layer (TL) prepared as a saturated solution of α-cyano-4-hydroxycinnamic acid (CHCA) matrix in acetonitrile (ACN). On the TL, we deposited 0.8 μL of peptide of interest. On each peptide spot, we added 0.8 μL of a matrix (CHCA and 2,5-Dihydroxybenzoic acid (DHB)) as final step. The samples were left to dry under the extractor hood at room temperature. 20 mg/mL CHCA solution was prepared in ACN and 5% FA (70:30, vol/vol). Final concentration of solutions was: 70% ACN and 1.5% FA. 20 mg/mL DHB solution in ACN and 0.1% trifluoroacetic acid (TFA)(70:30, vol/vol). Final concentration of solution was 70% ACN and 0.03% TFA. Then, CHCA and DHB were mixed in 1:1 ratio (Signor and Boeri Erba 2013). Next to the samples, we deposited 0.6 μL of a calibrant standard (peptide standard II, Bruker Daltonics) and then added 0.6 μL of the matrix solution. Regarding data analyzes, the spectra of intact peptides were processed using FlexAnalysis™ (version 3.4). Sophisticated Numerical Annotation Procedure detection algorithm was used for the identification of peaks with a threshold of 2 signal-to-noise ratio and a number maximal of 500 peaks detected. After this, it was applied automatic labelling and we manually reviewed the peak labelling.

### Purification of [Galβ1–4Xyl]-SDC3_short peptide

A large-scale synthesis reaction was conducted to generate a [Galβ1–4Xyl]-SDC3_short peptide for product inhibition experiments, similar to the process outlined above. In short, a 300 μL reaction mix was prepared containing 100 μM SDC3-short peptide, 1 mM UDP-Xyl, 1 mM UDP-Gal, 1 μM XylT1, and 8.4 μM β4GalT7 in the reaction buffer. This mixture was incubated in the dark for 16 h at room temperature. The glycosylated peptide was then purified by size exclusion chromatography and subsequently dialyzed against demineralized water, lyophilized and resuspended in demineralized water.

### In vitro glycosyltransferase activity of β4GalT7

Various sets of activity assays were conducted to investigate the β4GalT7 enzyme activity towards the SDC3_short peptide in more detail. All enzymatic reactions were carried out in the dark at room temperature, utilizing a reaction buffer comprising 25 mM MES at pH 6.5, 125 mM NaCl, 2.5 mM MgCl_2_, and 2.5 mM MnCl_2_. Two control reactions were performed, each containing 100 μM SDC3_short peptide, 1 μM XylT1, and either no or 1 mM UDP-Xyl.

#### UDP-gal concentration

Reaction mixtures of 20 μL were prepared, containing buffer, 100 μM SDC3_short peptide, 1 mM UDP-Xyl, 1 μM XylT1, and 8.4 μM β4GalT7, along with either 1 mM, 2 mM, or 5 mM UDP-Gal. After 16 h incubation, the reactions were stopped by transferring the mixture to - 20 °C and then analyzed by gel electrophoresis. The experiment was performed in triplicate.

#### Time-course

A reaction mixture of 20 μL, containing buffer, 100 μM SDC3_short peptide, 1 mM UDP-Xyl, 1 mM UDP-Gal, 1 μM XylT1, and 8.4 μM β4GalT7, was prepared. After incubation for 1, 2, 4, 6, 16, 48, and 72 h, 1 μL of the mixture was combined with 39 μL of 20% glycerol (v/v) containing phenol red and heat-inactivated at 85 °C for 10 min and then analyzed by gel electrophoresis. This time-course experiment was conducted in triplicate.

#### Product inhibition

A first 10 μL reaction mixture, consisting of buffer, 100 μM SDC3_short, 1 mM UDP-Xyl, 1 mM UDP-Gal, 1 μM XylT1, and 8.4 μM β4GalT7, was prepared. A second mix was set up similarly, but included 100 μM [Galβ1–4Xyl]-SDC3_short. After 16 h incubation, the reactions were stopped by storing the mix at −20 °C. Both reaction products and an additional control, consisting of a [Galβ1–4Xyl]-SDC3_short peptide sample, were observed by gel electrophoresis. This experiment was performed in duplicate.

#### Specificity of β3GalT6

20 μL reaction mixture were prepared containing 100 μM SDC3_short peptide, 1 mM UDP-Xyl and 1 μM XylT1. After 16 h incubation, 1 mM UDP-Gal, and 3.15 μM SUMO-β3GalT6 were added to the reaction, followed by an additional 24 h incubation. Reaction was stopped by placing mixture at −20 °C and then analyzed by gel electrophoresis. This experiment was realized in duplicate.

### In vitro glycosyltransferase activity of EXTL3

Peptides SDC3_short, SDC3_long, TGFBR3, CSF1_short, and CSF1_long, all carrying a tetrasaccharide [GlcAβ1–3Galβ1–3Galβ1–4Xyl]-linker, served as acceptor substrates to measure the GlcNAc-transferase activity of EXTL3. 10 μL of reaction mixture were prepared, containing 25 μM tetrasaccharide peptide, 3 mM UDP-GlcNAc, 1 μM EXTL3, and assay buffer (50 mM MES pH 6.5, 50 mM NaCl, 2.5 mM MgCl2, 2.5 mM MnCl2). Reactions were incubated at 37 °C in the dark and stopped after 0, 1, 2, 4 and 6 h by transferring 1 μL of reaction mix into 9 μL 20% glycerol (v/v) containing phenol red and heating for 10 min at 85 °C. The reactions were analyzed by gel electrophoresis using 25% polyacrylamide gels and band intensities quantified using Image Lab v.6.1. (Biorad). This experiment was realized in duplicate.

## Results

### Purification of the four linker-assembling glycosyltransferases

**Fig. 1:**
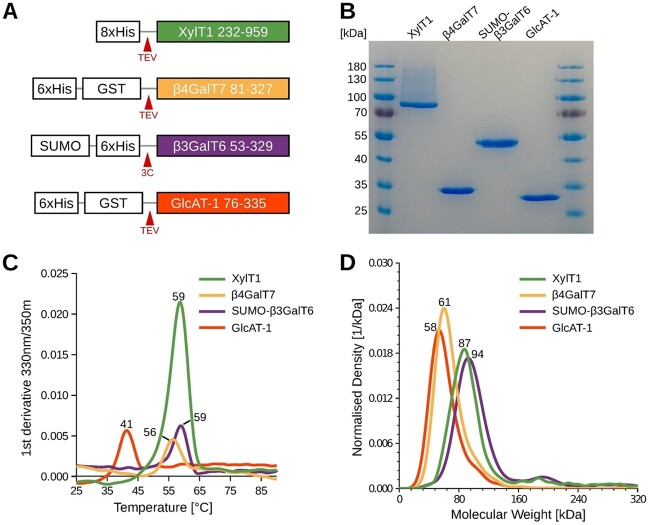
Purification and biophysical characterization of linker-synthesizing glycosyltransferases. A) Schematic representation of expression constructs featuring histidine purification tags (His) in conjunction with either glutathione S-transferase (GST) or SUMO fusion proteins. Tags can be cleaved using either tobacco etch virus (TEV) or 3C proteases. B) Coomassie-stained SDS-PAGE analysis of purified glycosyltransferases. Expected molecular sizes are 86 kDa for XylT1, 29 kDa for β4GalT7, 45 kDa for SUMO-β3GalT6, and 29 kDa for GlcAT-1. C) NanoDSF analysis of purified proteins, with melting temperatures depicted in the graph. D) Mass photometry analysis of purified glycosyltransferases, with determined molecular masses indicated in the graph.

To produce GAG-mimicking peptides containing the native tetrasaccharide linker, we expressed and purified the GTs responsible for the initial four steps in GAG biosynthesis. All four GTs were expressed as soluble proteins, omitting their N-terminal transmembrane anchoring helix to facilitate purification and subsequent enzymatic reactions (Fig. 1A). In a first attempt, we tried to recombinantly express the enzymes β4GalT7, β3GalT6 and GlcAT-1 in *E. coli* cells. However, due to low expression levels, we adapted a purification procedure for β3GalT6 from human embryonic kidney FreeStyle 293-F cells. XylT1 recombinant expression was performed in FreeStyle 293-F cells, following the methodology outlined in a prior study (Briggs and Hohenester 2018). XylT1 (residues 232–959) was expressed carrying an N-terminal 8xHistidine tag, followed by a Tobacco Etch Virus (TEV) cleavage site. β4GalT7 (residues 81–327) was expressed in *E. coli* as a fusion protein with an N-terminal 6xHistidine tag, a glutathione S-transferase tag (GST), and a TEV cleavage site. For β3GalT6 (residues 53–329) expression in FreeStyle 293-F cells, a construct was designed containing an N-terminal Small Ubiquitin-like Modifier (SUMO) tag, followed by a 6xHistidine tag and a 3C protease cleavage site. GlcAT-1 (residues 76–335) was expressed in *E. coli* using the same construct design as for β4GalT7. All proteins were purified using immobilized metal ion affinity chromatography (IMAC). The 6xHis-GST fusion tags of β4GalT7 and GlcAT-1 were removed by TEV protease treatment, followed by a second IMAC purification step. The SUMO-β3GalT6 fusion protein was not cleaved to improve protein stability. A final buffer exchange was performed using either dialysis or size exclusion chromatography. The obtained protein yields per liter of culture medium were 3.4 mg for XylT1, 0.9 mg for β4GalT7, 3.5 mg for SUMO-β3GalT6, and 0.35 mg for GlcAT-1. Purity of the samples was confirmed by SDS-PAGE analysis (Fig. 1B). For XylT1, in addition to the band at the expected molecular weight of 86 kDa, a high molecular weight smear was observed. One possible explanation could be that XylT1 undergoes various post-translational modifications.

### Biophysical characterization of linker enzymes

Next, we assessed the stability of the purified proteins using nano differential scanning fluorimetry (nanoDSF). The melting temperature (Tm), which indicates the temperature at which half of the protein is unfolded, is a key parameter for describing thermostability. XylT1, β4GalT7, and SUMO-β3GalT6 exhibited Tm values of 58.6 °C, 56.4 °C, and 58.8 °C, respectively, signifying a high level of thermal stability (Fig. 1C). Only one distinct melting point was observed for the SUMO-β3GalT6 fusion protein. Given that the SUMO tag lacks tryptophans and possesses only one tyrosine, the observed changes in the absorbance ratio likely stem from the unfolding of β3GalT6, which contains 9 tryptophans and 12 tyrosines. Notably, GlcAT-1 displayed a comparatively lower Tm value of 41.4 °C. The nanoDSF experiments demonstrate that the proteins remained stable in their respective storage buffers. However, it seems best to maintain the optimal incubation temperature for enzymatic reactions below 35 °C, particularly for GlcAT-1.

To gain further insight into sample homogeneity and the oligomeric structure of the four glycosyltransferases, we conducted mass photometry experiments. In all four cases, a prominent peak was observed, indicating both sample homogeneity and the presence of well-folded proteins in a defined oligomeric state. Specifically, XylT1 particles exhibited an average molecular weight of 87 kDa, aligning closely with the theoretical weight of 86 kDa for XylT1 in its monomeric state (Fig. 1D). On the other hand, the prevailing majority of particles for β4GalT7, SUMO-β3GalT6, and GlcAT-1 showed molecular weights of approximately 61 kDa, 94 kDa, and 58 kDa, respectively. These values correspond to the expected molecular weights for the proteins in their dimeric states. However, it is noteworthy that the anticipated molecular masses of monomeric β4GalT7 (29 kDa), SUMO-β3GalT6 (45 kDa), and GlcAT-1 (29 kDa) are in close proximity to/or fall below the detection limit of mass photometry. Consequently, it cannot be ruled out that these three proteins may also exist in a monomeric form in solution. However, since we observed only one peak for β4GalT7 and GlcAT-1 in size exclusion chromatography experiments, it is most likely that these proteins are in a dimeric state.

### Design of peptides mimicking GAG attachment sites

**Fig. 2:**
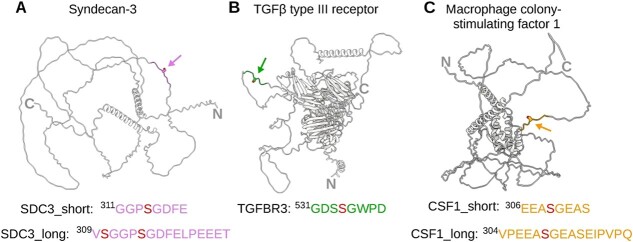
Mapping of GAG attachment sites on AlphaFold predicted proteoglycan structures. A) A cartoon representation of syndecan-3 in grey, with the presumable heparan sulfate attachment site and surrounding residues colored in rose. The amino acid sequences of the peptides used in this study, SDC3_short and SDC3_long, are indicated alongside. B) TGFβ type III receptor model with heparan sulfate attachment site and surrounding residues colored in green. The sequence of the TGFBR3 peptide is indicated. C) Cartoon representation of the chondroitin sulfate proteoglycan macrophage colony-stimulating factor 1 with its glycosaminoglycan attachment site and surrounding residues colored in orange. The amino acid sequences of the peptides CSF1_short and CSF1_long are indicated alongside.

The design of short peptide sequences was based on known sequences of proteoglycan core proteins, for which the precise GAG attachment sites have previously been experimentally determined (Noborn et al. 2022). This approach aimed to ensure that the chosen peptides would be readily recognized as substrates by the linker-synthesizing GTs. Specifically, two GAG attachment sites were selected based on heparan sulfate proteoglycans, namely syndecan-3 (SDC3) and TGFβ type III receptor (TGFBR3), as well as one derived from a chondroitin sulfate proteoglycan, macrophage colony-stimulating factor 1 (CSF1) (Carey et al. 1992; Chernousov and Carey 1993; Kimura et al. 1994; López-Casillas et al. 1994). To gain further insight into the positioning of the GAG attachment site, we examined the AlphaFold-predicted structures of the three proteoglycans (Jumper et al. 2021; Varadi et al. 2022). Upon comparison, an intriguing observation emerged: all three proteoglycans featured substantial unstructured regions (Fig. 2). Mapping of the serine residues serving as GAG attachment sites revealed their localization within these extensive unstructured regions, devoid of adjacent secondary structure elements.

In preparation for chemo-enzymatic synthesis, peptides of a length of eight amino acids were designed, including three preceding and four following the target serine residue. The first peptide, named SDC3_short and featuring the sequence ^311^GGPSGDFE was based on syndecan-3 (UniProt: O75056). It had been previously employed by Gao et al. for investigating XylT1 activity (Gao et al. 2021). Another peptide, TGFBR3, was derived from the TGFβ type III receptor (UniProt: Q03167) and harbors the sequence ^531^GDSSGWPD. A third peptide CSF1_short (^306^EEASGEAS) was based on the sequence of macrophage colony-stimulating factor 1 (UniProt: P09603). In order to study the effect of peptide lengths on enzymatic activity, we further designed longer peptides for syndecan-3 (SDC3_long, ^309^VSGGPSGDFELPEEET) and for the macrophage colony-stimulating factor 1 (CSF1_long, ^304^VPEEASGEASEIPVPQ).

### Enzymatic synthesis of tetrasaccharide-carrying linker peptides

**Fig. 3:**
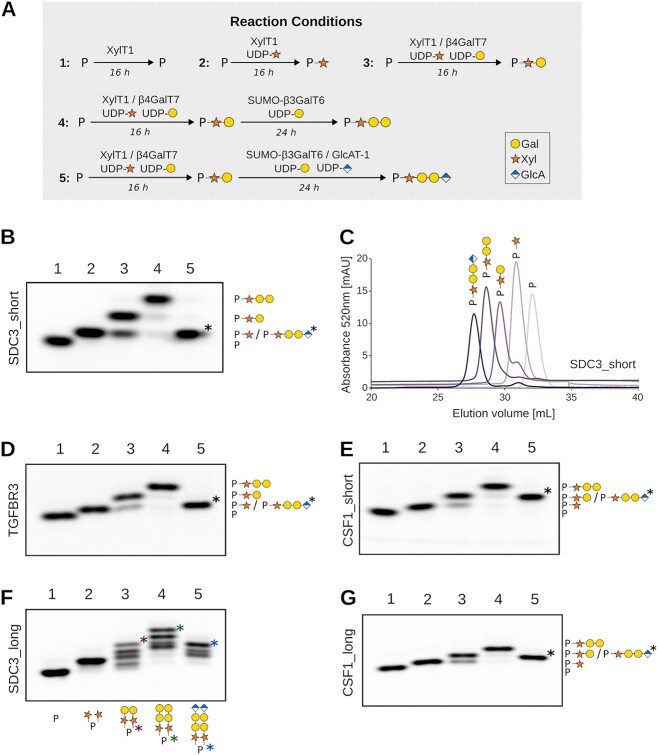
Step-wise addition of tetrasaccharide onto fluorescent peptides. A) Overview on the five enzymatic reactions performed to generate peptides carrying one, two, three or four glycan molecules. Monosaccharide symbols follow the SNFG (symbol nomenclature for Glycans) system (Varki et al. 2015). B) Analysis of glycan transfer onto peptide SDC3_short (^311^GGPSGDFE) upon incubation with linker enzymes and their corresponding substrates. Reactions were resolved by gel electrophoresis and fluorescent peptides were visualized using a fluorescence imager. The composition of reactions 1–5 is indicated in (A) and the identity of products on the right-hand side. C) Reaction mixtures 1–5 for peptide SDC3_short were further analyzed by size-exclusion chromatography. The graph depicts the overlay representation of the five elution profiles. D–G) Similar reactions were also conducted for peptide TGFBR3 (^531^GDSSGWP), CSF1_short (^306^EEASGEAS), SDC3_long (^309^VSGGPSGDFELPEEET) and CSF1_long (^304^VPEEASGEASEIPVPQ).

In order to produce peptides bearing the native tetrasaccharide [GlcAβ1–3Galβ1–3Galβ1–4Xyl], we conducted enzymatic reactions utilizing the four linker-synthesizing glycosyltransferases. For expedited and accurate analysis of the extension reactions, we designed fluorescently-labeled peptides with a N-terminal 5-Carboxytetramethylrhodamine (TAMRA) modification. The TAMRA fluorophore, with excitation and emission wavelengths of 541 nm and 568 nm respectively, enabled highly sensitive detection within the picomolar range. To monitor the addition of individual molecules, reactions were conducted in the presence of one, two, three, or all four linker enzymes (Fig. 3A).

The tetrasaccharide addition was performed in a two-step process. First, XylT1 and β4GalT7, along with their respective substrates UDP-Xyl and UDP-Gal, were incubated with the TAMRA-labeled peptides. Following a 16-hour incubation, SUMO-β3GalT6 and GlcAT-1 were added, along with UDP-GlcA and additional UDP-Gal. The reaction mixture was then incubated for another 24 h. The reaction products were separated using a 25% acrylamide gel and visualized through a fluorescence imaging system.

The successive addition of xylose, galactose, galactose, and glucuronic acid moieties onto the SDC3_short peptide (^311^GGPSGDFE) resulted in distinct band shifts (Fig. 3B, Supplementary Fig. 1A). Comparison of reaction 1 (lacking any UDP-sugar donor) with reaction 2 (containing XylT1 and UDP-Xyl) revealed a band shift, indicative of an increased molecular weight due to covalent glycan transfer. Notably, reaction 2 displayed only one band, suggesting complete reaction conversion. Following the addition of β4GalT7 and UDP-Gal (reaction 3), a further reduction in migration speed was observed, in line with the formation of a [Galβ1–4Xyl]-peptide product. However, the reaction was not entirely complete, as a band corresponding to the [Xyl]-peptide was also detected. An additional band shift was noted between reaction 3 and reaction 4, indicating the formation of a [Galβ1–3Galβ1–4Xyl]-peptide. Interestingly, the band corresponding to the intermediate [Xyl]-peptide was notably fainter in reaction 4 compared to reaction 3. The product of reaction 5, which included GlcAT-1 and UDP-GlcA, exhibited faster migration despite an increased molecular size. This observation can be attributed to the enhanced negative charge of the resulting [GlcAβ1–3Galβ1–3Galβ1–4Xyl]-peptide following glucuronic acid addition.

Chemo-enzymatic reaction products were further analyzed by size-exclusion chromatography. Each addition of a single sugar molecule led to a distinct shift in elution volume, further affirming the accurate addition of glycan moieties onto the SDC3_short peptide substrate (Fig. 3C). Parallel chemo-enzymatic reactions for the peptide TGFBR3 (^531^GDSSGWPD), CSF1_short (^306^EEASGEAS) and CSF1_long (^304^ VPEEASGEASEIPVPQ) yielded highly similar results. Reactions with SDC3_long (^309^VSGGPSGDFELPEEET), in contrast, led to a more complex band pattern (Fig. 3D–G, Supplementary Fig. 1B–E) due to the presence of two glycosaminoglycan attachment sites (^310^S and ^314^S). The HS attachment to ^310^S had not been described before, and its modification might only take place in the here described in vitro reaction conditions.

**Fig. 4:**
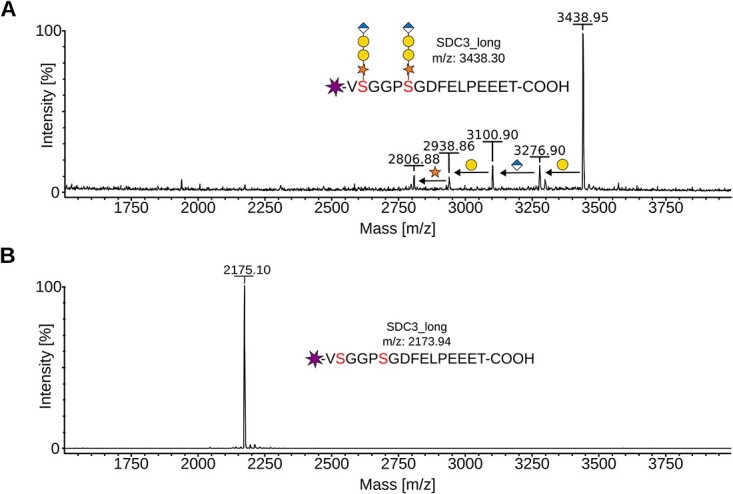
Mass spectrometry analysis of glycosylated SDC3_long peptide. A) Purified tetrasaccharide-carrying SDC3_long peptide was analyzed by MALDI-TOF. The major peak reflects the SDC3_long peptide carrying two [GlcAβ1–3Galβ1–3Galβ1–4Xyl]-glycans. Four additional minor signals correspond to less glycosylated peptides. The accurate mass difference between glycopeptides allows the assignment of distinct forms. Monosaccharide symbols follow the SNFG (symbol nomenclature for Glycans) system (Varki et al. 2015). B) Control MALDI-TOF spectra of non-glycosylated SDC3_long peptide.

Larger scale synthesis reactions were conducted to generate peptides with a tetrasaccharide linker. These tetrasaccharide peptides were subsequently purified by size exclusion chromatography to eliminate biosynthetic enzymes, UDP-sugar substrates and reaction products (Supplementary Fig. 2). It also resulted in a relatively homogeneous sample for peptide SDC3_long, carrying two tetrasaccharide linkers, as confirmed by mass spectrometry analysis (Fig. 4). Mass spectrometry analysis further revealed the presence of small amounts of a non-canonical trisaccharide linker [GlcAβ1–3Galβ1–4Xyl].

### Limiting factors in β4GalT7 activity

**Fig. 5:**
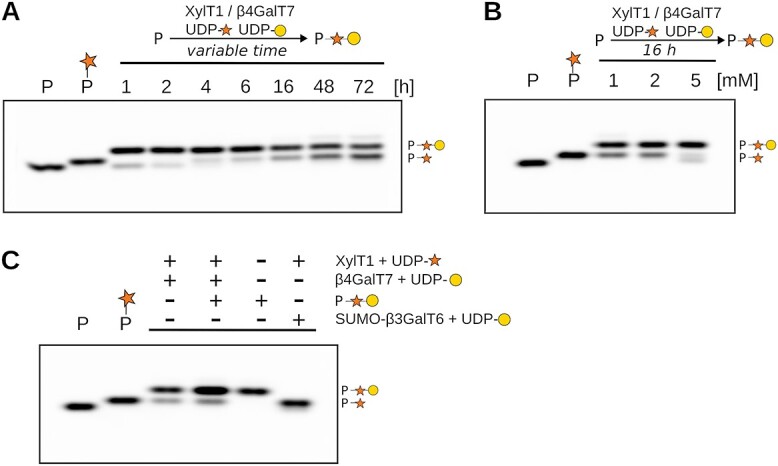
β4GalT7 activity towards SDC3_short peptide under various reaction conditions. A) Time-course experiment to follow galactose transfer by β4GalT7 onto SDC3_short peptide substrate. Reactions were stopped after 1, 2, 4, 6, 16, 48, and 72 h by heat-inactivation. B) Effect of donor substrate concentration on β4GalT7 activity was measured by varying UDP-Gal concentration from 1 mM to 2 mM and 5 mM. C) Potential influence of [Galβ1–4Xyl]-SDC3_short reaction product on β4GalT7 enzyme activity. Enzymatic reactions were carried out in the presence and absence of a purified [Galβ1–4Xyl]-SDC3_short peptide, which was added at the equimolar amount as the SDC3_short peptide. Purified [Galβ1–4Xyl]-SDC3_short alone served as negative control. Last lane shows reaction to study galactosyltransferase activity of β3GalT6 onto [Xyl]-SDC3_short peptide. Monosaccharide symbols follow the SNFG (symbol nomenclature for Glycans) system (Varki et al. 2015).

Intrigued by the fact that full conversion of the [Xyl]-peptides by β4GalT7 was reproducibly only observed, when the reaction was coupled to the subsequent step catalyzed by SUMO-β3GalT6, we set out to study the activity of β4GalT7 in more detail. When following galactose addition on the SDC3_short peptide by β4GalT7 over 72 h, we found that the amount of generated [Galβ1–4Xyl]-peptide increased over the first 4 h and then decreased at longer incubation times (Fig. 5A, Supplementary Fig. 3A). To test whether the UDP-galactose substrate was limiting in the transfer reaction, we varied its concentration from 1 mM to 5 mM. Increasing the donor substrate concentration did not significantly increase reaction completeness (Fig. 5B, Supplementary Fig. 3B). Even more, at a concentration of 5 mM UDP-galactose, a faint band for the nonglycosylated peptide appeared, suggesting that higher UDP-galactose concentrations could inhibit XylT1 activity. Another possible explanation for observing reaction completeness only in the presence of SUMO-β3GalT6, is that the latter removes the [Galβ1–4Xyl]-peptide product. To explore the potential inhibition of β4GalT7 by its own product, we purified [Galβ1–4Xyl]-SDC3_short peptide. The activity of β4GalT7 in presence of its reaction product was found to be lower, as the non-converted [Xyl]-peptide increased by around 60% (Fig. 5C). Of note, SUMO-β3GalT6 did not extend the [Xyl]-SDC3_short peptide in the absence of β4GalT7 activity (Fig. 5C, Supplementary Fig. 3C).

### Role of core protein sequence in the divergent step of GAG biosynthesis

**Fig. 6:**
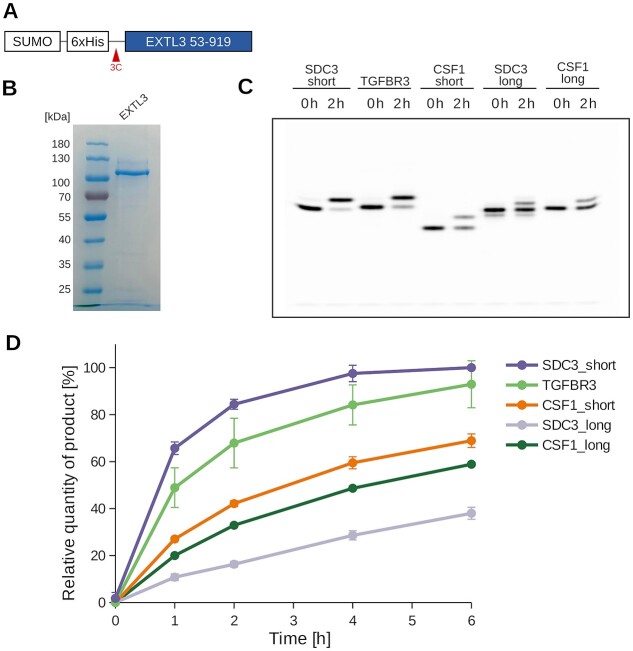
Substrate specificity tests using recombinant EXTL3. A) Schematic representation of the recombinant EXTL3 construct. The N-terminal anchoring helix is omitted, and the construct features a cleavable N-terminal SUMO fusion protein followed by a 6xHistidine purification tag. B) Coomassie-stained SDS-PAGE analysis of purified EXTL3, with a theoretical molecular weight of 99 kDa. (C) Analysis of EXTL3 activity towards five distinct tetrasaccharide peptides: SDC3_short (^311^GGPSGDFE), TGFBR3 (^531^GDSSGWP), CSF1_short (^306^EEASGEAS), SDC3_long (^309^VSGGPSGDFELPEEET) and CSF1_long (^304^VPEEASGEASEIPVPQ). Reactions after 0 and 2 h incubation time were analyzed by gel electrophoresis (see Supplementary Fig. 4 for all reaction points). D) GlcNAc transfer by EXTL3 onto the five different peptides was quantified (see polyacrylamide gels in Supplementary Fig. 4). Product formation was followed over 6 h. For the SDC3_long peptide, which harbors two tetrasaccharides, the addition of either one or two GlcNAc moieties could be observed. For calculating the conversion rate, the addition of at least one GlcNAc residue was considered.

The biosynthesis of various glycosaminoglycans commences with the assembly of the tetrasaccharide linker. The addition of the fifth sugar molecule marks a pivotal divergence point in the biosynthetic pathways: the incorporation of a GlcNAc residue initiates HS/Hep production, while the addition of GalNAc leads to the formation of a CS/DS chain. To gain a deeper insight into the regulatory mechanisms governing this critical step, we aimed on elucidating the substrate specificity of EXTL3, the enzyme responsible for transferring GlcNAc onto the tetrasaccharide linker. We successfully expressed and purified EXTL3 (Fig. 6A and B, Supplementary Fig. 4). SDS-PAGE analysis suggested that EXTL3 protein was highly pure and that it carries several post-translational modifications, e.g. *N-*glycans, as its apparent molecular mass was around 10 kDa higher than expected.

Next, we investigated the GlcNAc transfer activity of EXTL3 towards five tetrasaccharide peptides derived from the HSPGs syndecan-3 (SDC3_short and SDC3_long) and TGFβ type III receptor (TGFBR3), as well as the CSPG macrophage colony-stimulating factor 1 (CSF1_short and CSF1_long). EXTL3-catalyzed GlcNAc addition onto the tetrasaccharide peptides was followed for 1, 2, 4 and 6 h (Fig. 6C and D, Supplementary Fig. 5).

Highest reaction completeness after 2 h was observed for the SDC3_short peptide (84%). The remaining tetrasaccharide peptides were converted with decreasing efficiency: TGFBR3 (68%) > CSF1_short (42%) > CSF1_long (33%) > SDC3_long (16%) (Fig. 6C and D, Supplementary Fig. 5). This suggests that the short 8-amino acid peptides derived from HSPGs (SDC3_short, TGFBR3) are better substrates for EXLT3 compared to the one originating from CSGPs (CSF1_short). The longer peptides showed lower conversion rates than their shorter counterparts. For SDC3_long, which carries two tetrasaccharide linkers, we could see the addition of either one or two GlcNAc residues. However, the band corresponding to the addition of two GlcNAc residues was barely detectable.

## Discussion

GAGs are essential linear polysaccharide chains found on cell surfaces and in the extracellular matrix. Among these, HS and CS are prominent, both attached to the core protein of proteoglycans through a shared tetrasaccharide linker consisting of glucuronic acid, galactose, galactose, and xylose. This linker is meticulously constructed by a group of glycosyltransferases localized in the Golgi apparatus. The addition of the fifth sugar, either GlcNAc or GalNAc, marks a pivotal point where HS and CS chains diverge, each serving distinct biological functions. The regulatory mechanisms governing this crucial step remain elusive. It is tempting to speculate that, considering glycosaminoglycan biosynthesis occurs post-translationally in the Golgi apparatus, the protein’s amino acid sequence or its three-dimensional structure may encode the information dictating its fate as either an HSPG or CSPG. To explore this hypothesis, we established a methodology for generating fluorescent peptides carrying the shared GAG linker.

Peptides were designed based on key proteoglycans—syndecan-3 and TGFβ type III receptor, emblematic of HSPGs, and macrophage colony-stimulating factor 1, a representative of CSPGs. Structural analysis using AlphaFold predictions unveiled that all peptides are situated within highly flexible regions (Fig. 2). It is noteworthy that while AlphaFold may not definitively confirm protein disorderliness, it does suggest regions amenable to structural flexibility (Ruff and Pappu 2021; Guo et al. 2022). These unstructured regions may adopt a more ordered conformation within the cell through interactions with other proteins or GAG chains (Gondelaud et al. 2021). The high flexibility surrounding the GAG attachment site potentially confers an advantage for post-translational glycan moiety addition, likely by facilitating access to catalytic sites (Bludau et al. 2022).

The designed peptides were readily accepted by the linker enzymes as substrates, with most reactions reaching completion (Fig. 3). However, the β4GalT7 reaction, responsible for generating [Galβ1–4Xyl]-peptide, displayed partial completeness, leaving a discernible [Xyl]-peptide band on the gel. Interestingly, this band diminished when β3GalT6 was added after 16 h to the XylT1 and β4GalT7 containing reaction. To eliminate the possibility that this effect was merely a result of extended incubation, a time-course experiment was conducted. Surprisingly, we observed a decrease in the quantity of the generated [Galβ1–4Xyl]-peptide after an initial accumulation, concurrently with the accumulation of the [Xyl]-peptide intermediate (Fig. 5A). One plausible explanation is that galactose undergoes hydrolysis from the generated [Galβ1–4Xyl]-peptide, and with prolonged incubation times, the UDP-Gal substrate is depleted or β4GalT7 looses activity, preventing the generation of new [Galβ1–4Xyl]-peptide. The hydrolysis is likely enzyme-assisted, given that the [Galβ1–4Xyl]-peptide remains stable upon heat inactivation of the sample. Considering both the hydrolysis of [Galβ1–4Xyl]-peptide and the observed product inhibition affecting β4GalT7 activity (Fig. 5C), we propose that β3GalT6 enhances the efficiency of β4GalT7 by eliminating its [Galβ1–4Xyl]-peptide product. It should be mentioned, that β4GalT7 and GlcAT-1 were expressed in *E. coli* cells, lacking their native post-translational modifications, including *N-*glycans. This absence could potentially impact their stability, activity, and specificity. Another possibility, which remains to be investigated, is a cooperative function between β3GalT6 and β4GalT7, possibly facilitated by functional or physical complexes (Kellokumpu et al. 2016; Li and Kusche-Gullberg 2016). It should be noted that when XylT1, β4GalT7, and β3GalT6 were introduced simultaneously in the reaction, none of the sugar transfers were completed. This resulted in a very low final proportion of peptide with [Galβ1–3Galβ1–4Xyl] (results not shown). This phenomenon might be attributed to the potential formation of a non-canonical Gal-Xyl-peptide (Persson et al. 2019; Delbaere et al. 2020) or the competition between β4GalT7 and β3GalT6 for the UDP-Gal substrate.

Linker generation was previously shown to be fine-tuned by xylose phosphorylation/dephosphorylation (Koike et al. 2014). Phosphorylation by the enzyme FAM20B most likely occurs after [Galβ1–4Xyl]-peptide formation by β4GalT7 (Gulberti et al. 2005) and was further shown to enhance β3GalT6 activity (Wen et al. 2014; Sammon et al. 2023). Of note, we could observe full completeness for the β3GalT6-catalyzed reaction in the absence of phosphorylation under our chosen reaction conditions, which included sufficient amounts of recombinant enzyme and relatively long incubation times (Fig. 3).

Next, we examined the substrate specificity of EXTL3 using purified tetrasaccharide peptides. The most rapid GlcNAc addition was observed for syndecan-3-derived peptide SDC3_short (HSPG), followed by the TGFβ type III receptor-derived peptide TGFBR3 (HSPG) and the macrophage colony-stimulating factor 1 peptide CSF1_short (CSPG) (Fig. 6). Notably, this substrate preference of EXTL3 for peptides derived from HSPGs over CSPGs has recently been reported by Sammon et al. (Sammon et al. 2023). To test the effect of peptide length on EXTL3 activity, we designed peptides SDC3_long and CSF1_long that contained two additional amino acid residues at the N-terminal end and six additional residues at the C-terminal end. Both peptides were converted less efficiently compared to their shorter versions, with a larger difference observed between peptides SDC3_short and SDC3_long. The SDC3_long peptide contains three additional Glu residues compared to SDC3_short and it has been recently proposed by Sammon et al. that acid residues bind to a basic surface patch in EXTL3. We would have thus expected an increased affinity for the peptide SDC3_long over SDC3_short. One possible explanation for a reduced activity of EXTL3 for the SDC3_long peptide could be that it carries two glycosylation sites, of which one has not been observed before, and that the additional tetrasaccharide blocks access to the active site in EXTL3. Our findings suggest that the sequence in close proximity to the glycosaminoglycan attachment site contains the information recognized by EXTL3. Therefore, EXTL3 emerges as a key player in the decision-making process during glycosaminoglycan chain biosynthesis.

Nevertheless, CSPG peptides were also processed by EXTL3 and the differences in conversion between the different peptides do not seem sufficient to explain the degree of specificity during glycosaminoglycan chain addition observed in the cell. We cannot exclude that this is due to our in vitro reaction conditions or the lack of xylose phosphorylation, which was shown to enhance EXTL3 activity (Sammon et al. 2023). Additionally, the absence of the N-terminal transmembrane anchoring helix in the EXTL3 construct could impact its activity. On the other hand, it might also hint that regulation of the divergent step is more intricate and might involve additional cellular factors or larger parts of the PG. It could also represent a potential rescue mechanism, in which EXTL3 adds HS chains onto CSPG core proteins in the absence of enzymes initiating CS biosynthesis (Takeuchi et al. 2013). These intricate regulatory mechanisms warrant further investigation, including substrate specificity assessments of CSGALNACT-1/CSGALNACT-2, competition assays between CSGALNACTs and EXTL3, and testing peptides/core proteins of varying lengths. Furthermore, the influence of modifications of the tetrasaccharide linker, such as phosphorylation of the Xyl residue, can be explored as additional regulatory mechanisms (Koike et al. 2014; Wen et al. 2014).

In conclusion, our established system for generating fluorescent peptides provides an efficient means to characterize different peptide substrates in the divergent step of GAG biosynthesis. This methodology paves the way for future research in unraveling the intricate regulatory network governing GAG biosynthesis, shedding light on its fundamental biological significance.

## Supplementary Material

LinkerSynthesis_revision_SM_26Feb_final_cwae016

EXTL3_activity_raw_data_cwae016

NanoDSF_raw_data_cwae016

Mass_photometry_raw_data_cwae016

## Data Availability

Source data are provided with this paper.

## CRediT author statement

Marie Bourgeais (Data curation [lead], Formal analysis [equal], Investigation [lead], Methodology [lead], Visualization [equal], Writing—original draft [equal]), Farah Fouladkar (Data curation [supporting], Investigation [supporting], Methodology [supporting], Writing—review & editing [supporting]), Margot Weber (Investigation [supporting], Methodology [supporting]), Elisabetta Boeri-Erba (Investigation [supporting], Methodology [supporting], Writing—review & editing [supporting]), and Rebekka Wild (Conceptualization [lead], Data curation [equal], Formal analysis [equal], Funding acquisition [lead], Investigation [supporting], Methodology [supporting], Project administration [lead], Resources [lead], Supervision [lead], Validation [lead], Visualization [equal], Writing—original draft [equal], Writing—review & editing [equal]).
