# Supplementary material for: Chemo-enzymatic synthesis of tetrasaccharide linker peptides to study the divergent step in glycosaminoglycan biosynthesis
Source: Glycobiology. 2024 Feb 24;34(5):cwae016. doi: 10.1093/glycob/cwae016 (PMC11031135; doi:10.1093/glycob/cwae016)
Supplement: LinkerSynthesis_revision_SM_26Feb_final_cwae016 [file linkersynthesis_revision_sm_26feb_final_cwae016.pdf]

Supplementary Information

Chemo-enzymatic synthesis of tetrasaccharide linker peptides to study the divergent step in glycosaminoglycan biosynthesis

Marie Bourgeais[1], Farah Fouladkar[1], Margot Weber[1], Elisabetta Boeri-Erba[1], Rebekka Wild[1,#]

[1]Institut de Biologie Structurale, UMR 5075, University Grenoble Alpes, CNRS, CEA, 38000 Grenoble, France.

[#]Correspondence: rebekka.wild@ibs.fr

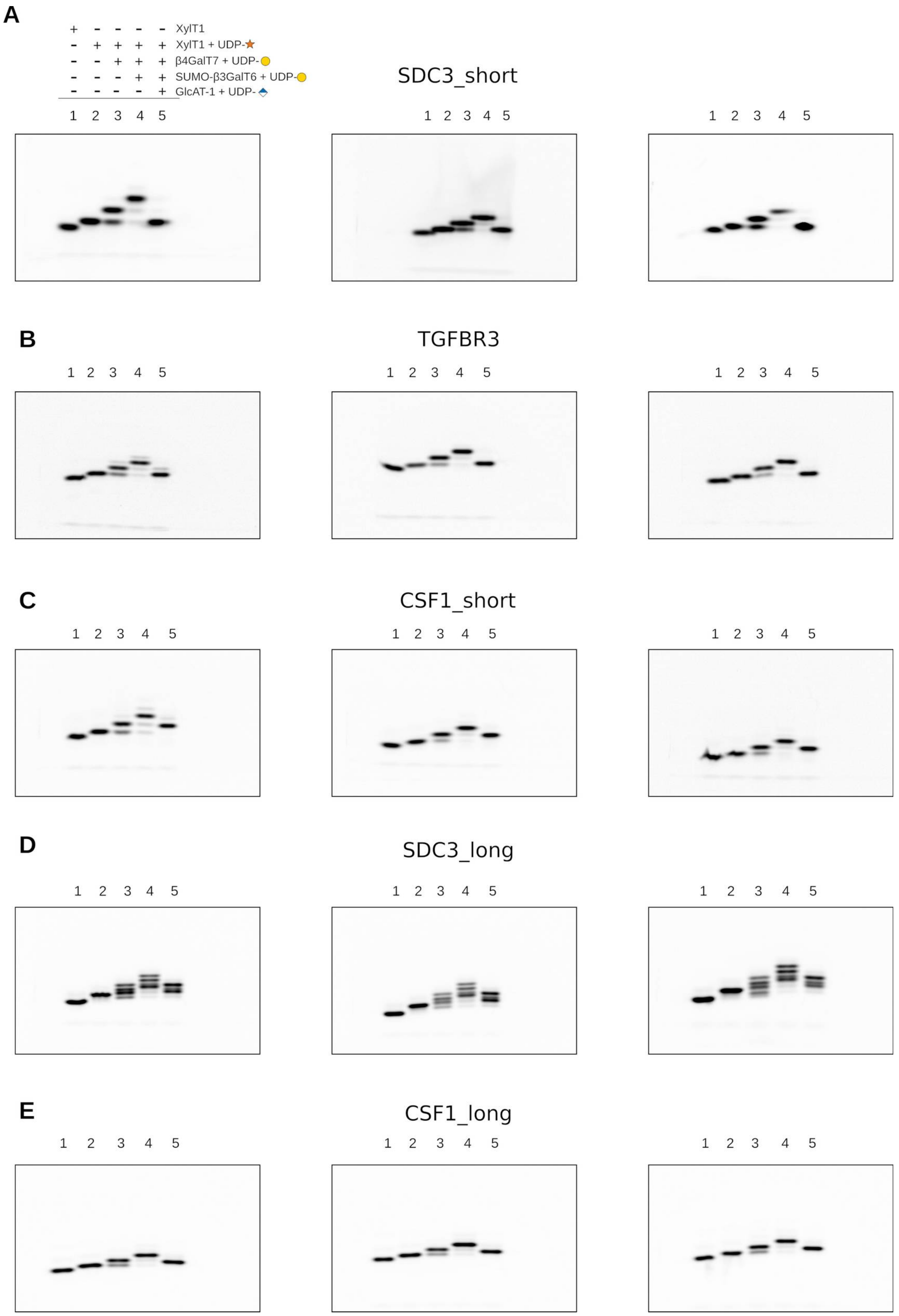

Supplementary Figure 1: Uncropped gels for step-wise addition of tetrasaccharide onto peptides

(**A-E**) Analysis of glycan transfer onto peptides SDC3_short (^{311}GGPSGDFE), TGFBR3 (^{531}GDSSGWP), CSF1_short (^{306}EEASGEAS), SDC3_long (^{309}VSGGPSGDFELPEEET) and CSF1_long (^{304}VPEEASGEASEIPVPQ) using 25% polyacrylamide gels and fluorescent signal detection at 546 nm. Experiments performed in triplicate.

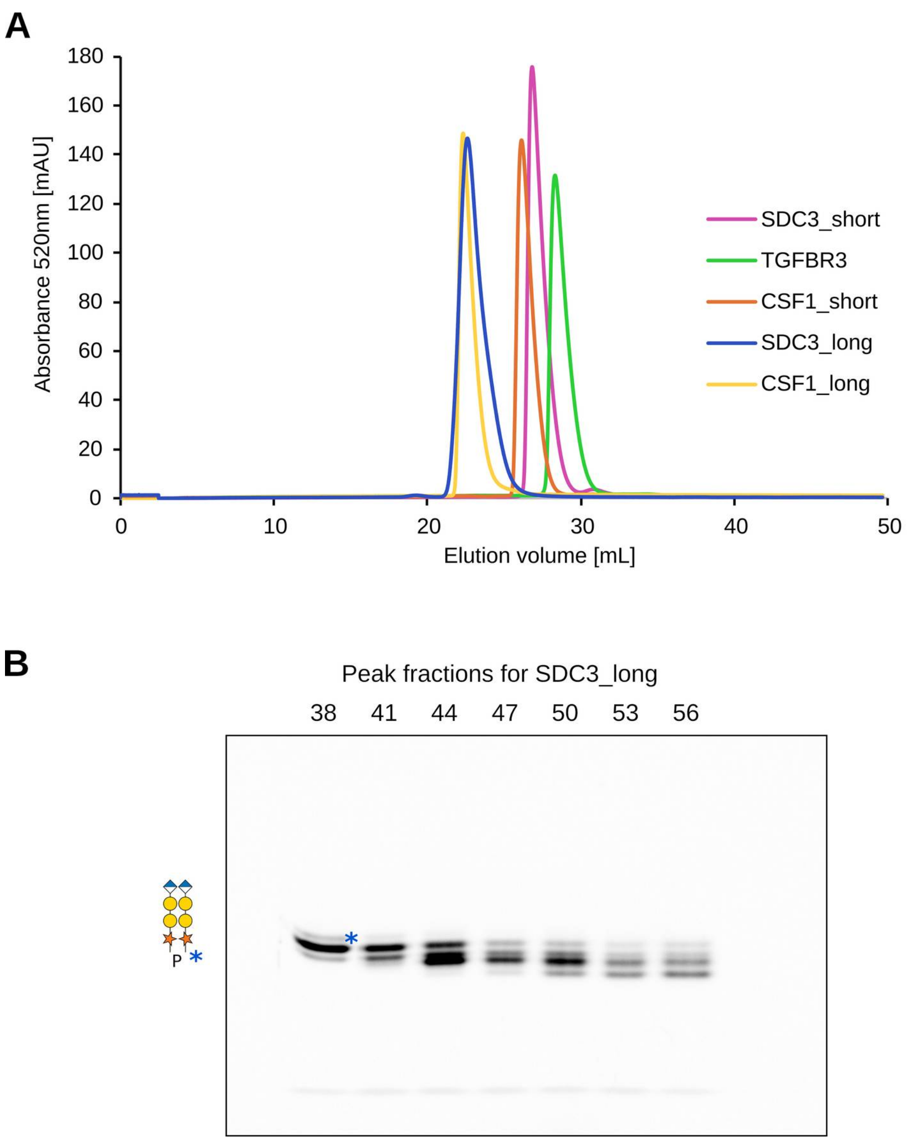

Supplementary Figure 2: Larger scale tetrasaccharide peptide synthesis

(**A**) A [GlcAβ1–3Galβ1–3Galβ1–4Xyl] tetrasaccharide was added onto peptides SDC3_short (^{311}GGPSGDFE), TGFBR3 (^{531}GDSSGWP), CSF1_short (^{306}EEASGEAS), SDC3_long (^{309}VSGGPSGDFELPEEET) and CSF1_long (^{304}VPEEASGEASEIPVPQ) using recombinant linker enzymes. Reaction products were purified by size-exclusion chromatography.

(**B**) Peak fractions of the glycosylated SDC3_long peptide were analyzed on a 25 % polyacrylamide gel. The blue asterisk indicates lane, which contains predominantely peptide harbouring two tetrasaccharide linkers, as analyzed by MALDI-TOF (see Figure 4).

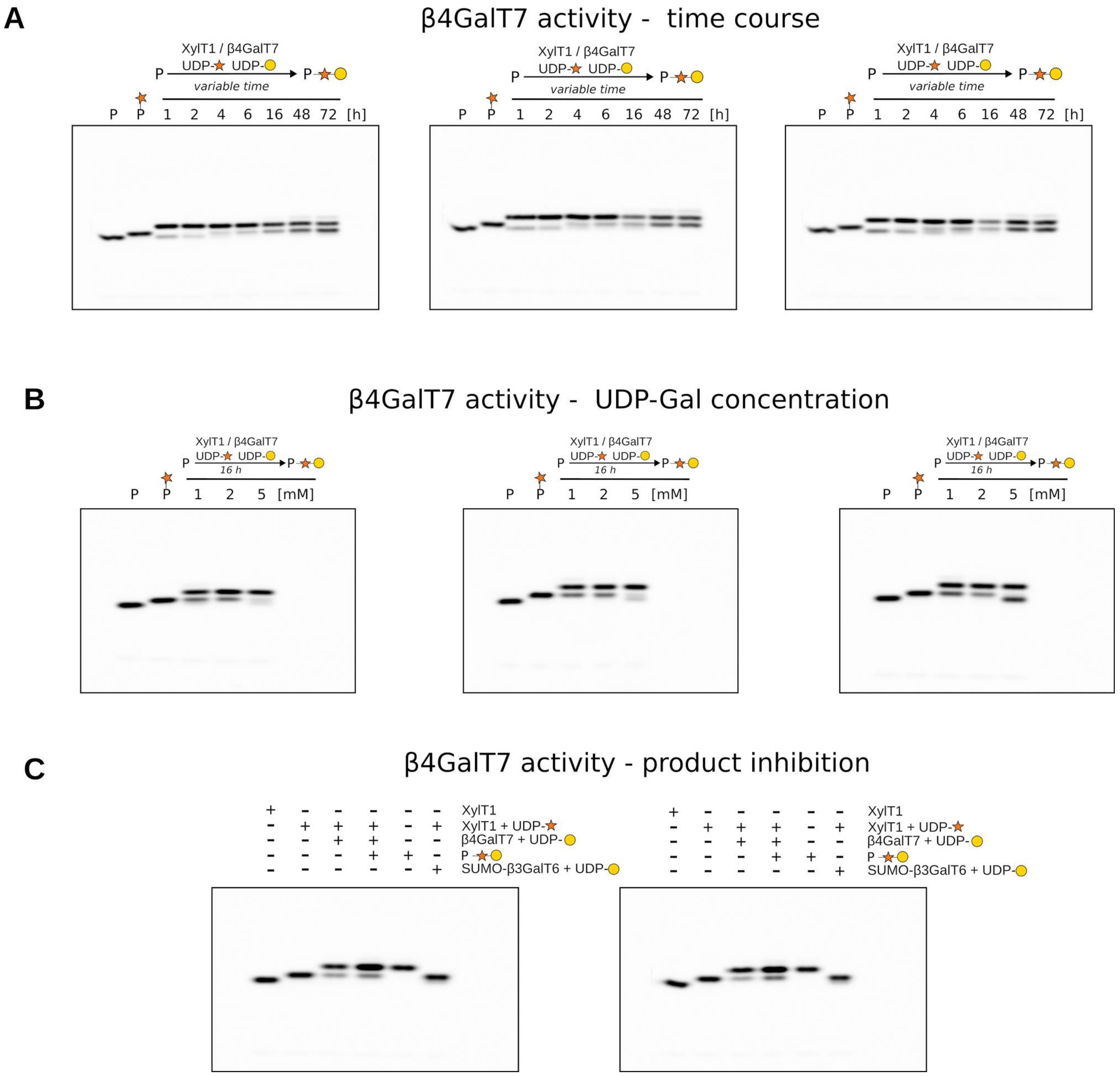

Supplementary Figure 3: Uncropped gels for tests on β4GalT7 activity

(**A**) Time-course experiment to follow galactose transfer by β4GalT7 onto SDC3_short peptide substrate. Reactions were stopped after 1, 2, 4, 6, 16, 48, and 72 h by heat-inactivation.

(**B**) Effect of donor substrate concentration on β4GalT7 activity was measured by varying UDP-Gal concentration from 1 mM to 2 mM and 5 mM.

(**C**) Potential influence of [Galβ1–4Xyl]-SDC3_short reaction product on β4GalT7 enzyme activity. Enyzmatic reactions were carried out in the presence and absence of a purified [Galβ1–4Xyl]-SDC3_short peptide, which was added at the equimolar amount as the SDC3_short peptide. Purified [Galβ1–4Xyl]-SDC3_short alone served as negative control. Last lane shows reaction to study galactosyltransferase activity of β3GalT6 onto [Xyl]-SDC3_short peptide.

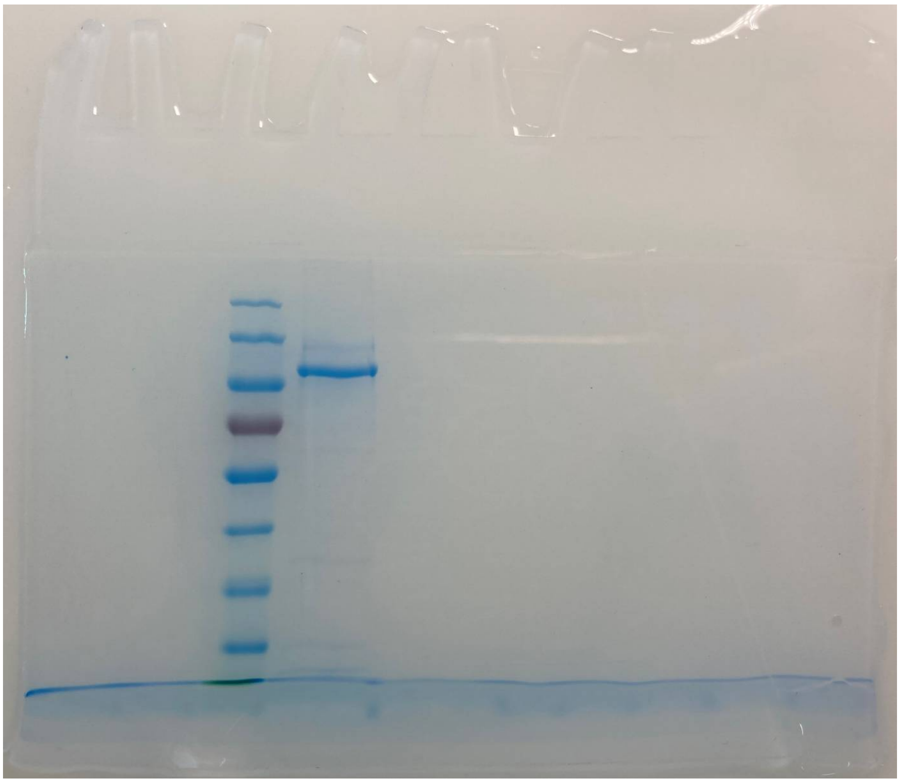

Supplementary Figure 4: Uncropped Coomassie blue stained SDS-PAGE gel from Figure 6B showing purified EXTL3 protein

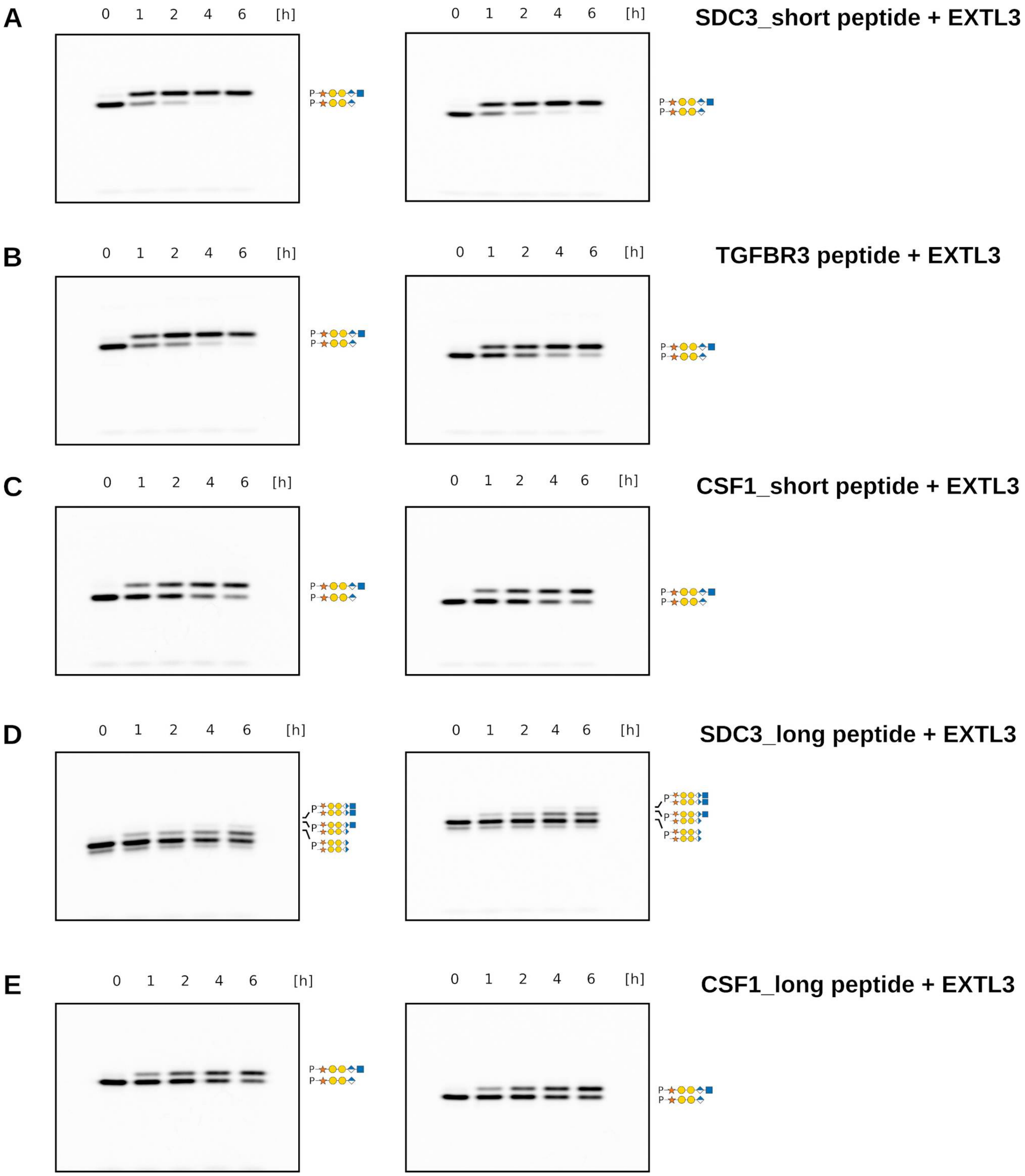

Supplementary Figure 5: Uncropped gels for EXTL3 activity assays using tetrasaccharide peptide substrates

GlcNAc transfer onto SDC3_short (^{311}GGPSGDFE), TGFBR3 (^{531}GDSSGWP), CSF1_short (^{306}EEASGEAS), SDC3_long (^{309}VSGGPSGDFELPEEET) and CSF1_long (^{304}VPEEASGEASEIPVPQ) was followed over 6 h. Reactions were analyzed by 25% polyacrylamide gel electrophoresis and fluorescence imaging. The experiments were performed in duplicate.
